# RAPID: an interactive R/Shiny platform for end-to-end 16S rRNA and ITS amplicon sequence analysis using DADA2

**DOI:** 10.1093/bioinformatics/btag688

**Published:** 2026-09-15

**Authors:** Beant Kapoor, Melissa A Cregger, Jill Hamilton, Alayna Mead, Priya Ranjan

**Affiliations:** Biosciences Division, Oak Ridge National Laboratory, Oak Ridge, TN, United States; Biosciences Division, Oak Ridge National Laboratory, Oak Ridge, TN, United States; College of Agricultural Sciences, Pennsylvania State University, University Park, PA, United States; College of Agricultural Sciences, Pennsylvania State University, University Park, PA, United States; Biosciences Division, Oak Ridge National Laboratory, Oak Ridge, TN, United States

## Abstract

**Motivation:**

Amplicon sequencing of 16S rRNA and internal transcribed spacer (ITS) gene regions is the most widely used approach for characterizing bacterial and fungal communities. The DADA2 pipeline has become the standard for inferring amplicon sequence variants (ASVs), offering single-nucleotide resolution over traditional OTU clustering. However, executing the full DADA2 workflow requires R programming proficiency and manual coordination of multiple sequential steps, presenting a substantial barrier for researchers in clinical, environmental, and agricultural sciences who lack computational training.

**Results:**

We present RAPID (R-based Amplicon Pipeline for Interactive DADA2), a pair of R/Shiny applications providing complete graphical user interfaces for 16S rRNA and ITS amplicon analysis. The 16S application implements a 10-step guided workflow from raw paired-end FASTQ files through quality filtering, denoising, paired-read merging, chimera removal, SILVA-based taxonomy assignment, phyloseq construction with data transformation (rarefaction, relative abundance, or CLR), visualization (rarefaction curves, alpha diversity, NMDS, PCoA, abundance), PERMANOVA, and ANCOM-BC2 differential abundance analysis. The ITS application extends this to 11 steps, adding automated primer removal via cutadapt with support for multiple primers and length-variable amplicons, and uses the UNITE database for fungal taxonomy. Both applications feature asynchronous background processing, session persistence, real-time progress monitoring, publication-ready figure export at 300 DPI, and comprehensive CSV/PNG result downloads.

**Availability:**

Raw 16S rRNA amplicon sequences are available in the NCBI Sequence Read Archive under BioProject accession PRJNA1499817. RAPID is freely available at https://github.com/beantkapoor786/RAPID and archived at 10.5281/zenodo.21628564. Both applications can be installed locally on any system with R (≥4.0) and run as local web applications.

## 1 Introduction

High-throughput amplicon sequencing of marker genes has become the cornerstone of microbial ecology (Caporaso *et al*. 2012, Hugerth and Andersson 2017). For bacteria and archaea, the 16S ribosomal RNA gene is the standard target, while the internal transcribed spacer (ITS) region is used for fungal community profiling (Schoch *et al*. 2012). The field has seen a paradigm shift from operational taxonomic unit (OTU) clustering at 97% sequence identity toward exact amplicon sequence variants (ASVs), which provide single-nucleotide resolution and improved reproducibility across studies (Callahan *et al*. 2017).

The DADA2 algorithm has emerged as one of the most widely adopted tools for ASV inference from Illumina amplicon data (Callahan *et al*. 2016). By modeling sequencing run-specific error rates, DADA2 distinguishes true biological sequences from artifacts without arbitrary similarity thresholds. DADA2 is available as an R/Bioconductor package and can also be accessed through the QIIME 2 platform (Bolyen *et al*. 2019). DADA2 supports both 16S and ITS workflows, though the ITS pipeline requires additional steps for primer removal due to the highly variable length of ITS amplicons (200–600 bp), which precludes fixed-length truncation and necessitates external tools such as cutadapt (Martin 2011).

Despite the methodological advances offered by DADA2, a significant accessibility gap persists. Executing the complete pipeline requires users to write R scripts, manage package dependencies across CRAN and Bioconductor, configure platform-specific parallelization settings, manually inspect quality profiles, download and manage taxonomy reference databases, and construct phyloseq objects for downstream ecological analysis (McMurdie and Holmes 2013). For ITS workflows, the additional complexity of primer removal with cutadapt, handling variable-length amplicons, and using the UNITE database further raises the barrier (Nilsson *et al*. 2019).

Several tools have addressed parts of this challenge. QIIME 2 provides a comprehensive command-line framework but requires terminal proficiency. Shiny-phyloseq offers interactive visualization of processed microbiome data but does not handle upstream sequence processing (McMurdie and Holmes 2015). MOCHI provides a GUI for amplicon analysis but uses a different processing engine (Zheng *et al*. 2022). Galaxy-based workflows offer web-based interfaces but require server infrastructure (Batut *et al*. 2018). Tourmaline provides an end-to-end DADA2-based workflow implemented in Snakemake; however, this command-line solution requires familiarity with bioinformatics environments and lacks the interactive, graphical interface that RAPID provides (Thompson *et al*. 2022). More broadly, none of these tools offer a self-contained, locally installable graphical interface that spans the entire workflow, from raw FASTQ files to statistical testing and publication-ready visualizations, using the DADA2 algorithm for both bacterial (16S) and fungal (ITS) marker genes.

Here, we present RAPID, a pair of R/Shiny applications that wrap the complete DADA2 pipeline and downstream analysis into guided, point-and-click graphical user interfaces for both 16S rRNA and ITS amplicon sequencing data. The applications share a common architectural framework but are tailored to the specific requirements of each marker gene, including ITS-specific primer removal and the absence of fixed-length truncation.

## 2 Results

### 2.1 Shared application architecture

Both RAPID applications were developed using the R Shiny framework (Jia *et al*. 2022) and share a common architecture featuring: (i) a step-by-step guided workflow with contextual controls and result summaries at each stage; (ii) asynchronous computation via callr background subprocesses, enabling multicore DADA2 parallelism while maintaining full interface responsiveness; (iii) automatic session persistence to the data directory; (iv) a modern dark-themed interface with real-time progress indicators; and (v) publication-ready figure export at 300 DPI. All computationally intensive steps (quality filtering, error learning, dereplication, chimera removal, taxonomy assignment, and ANCOM-BC2) are offloaded to background R subprocesses, resolving the fundamental incompatibility between DADA2’s fork-based multiprocessing and Shiny’s reactive framework. Because fork-based parallelism is not natively available on Windows, RAPID automatically detects the operating system at runtime and enables multithreading on macOS and Linux while falling back to single-threaded processing on Windows. Users on Windows should expect longer runtimes for computationally intensive steps; results remain identical across platforms.

### 2.2 The 16S rRNA application (10 steps)

The 16S application implements a 10-step workflow: (1) Setup and file detection with auto-identification of forward/reverse read patterns and configurable sample name extraction (Fig. 1); (2) quality profile visualization; (3) quality filtering and trimming with all DADA2 parameters exposed (truncLen, maxEE, truncQ, maxN, rm.phix) (Fig. S1, available as supplementary data at *Bioinformatics* online); (4) error rate learning and sequence dereplication (Fig. S2, available as supplementary data at *Bioinformatics* online); (5) paired-read merging and chimera removal with a read-tracking table (Supplementary Figs S3 and S4, available as supplementary data at *Bioinformatics* online); (6) taxonomy assignment using SILVA v138.1 with support for both the naive Bayesian classifier and IdTaxa via DECIPHER (Wang *et al*. 2007, Quast *et al*. 2013, Murali *et al*. 2018) (Fig. S5, available as supplementary data at *Bioinformatics* online); (7) phyloseq object construction with metadata upload, variable type selection, and data transformation (rarefaction, relative abundance, or centered log-ratio via the microbiome package) (Fig. S6, available as supplementary data at *Bioinformatics* online); (8) downloaded plots of rarefaction curves, alpha diversity boxplots, and NMDS/PCoA ordination with 95% confidence ellipses (Fig. S7, available as supplementary data at *Bioinformatics* online); (9) PERMANOVA with user-specified formula, betadisper homogeneity testing, and pairwise comparisons with Bonferroni correction (Fig. S8, available as supplementary data at *Bioinformatics* online); and (10) ANCOM-BC2 differential abundance analysis (Fig. S9, available as supplementary data at *Bioinformatics* online).

**Figure 1:**
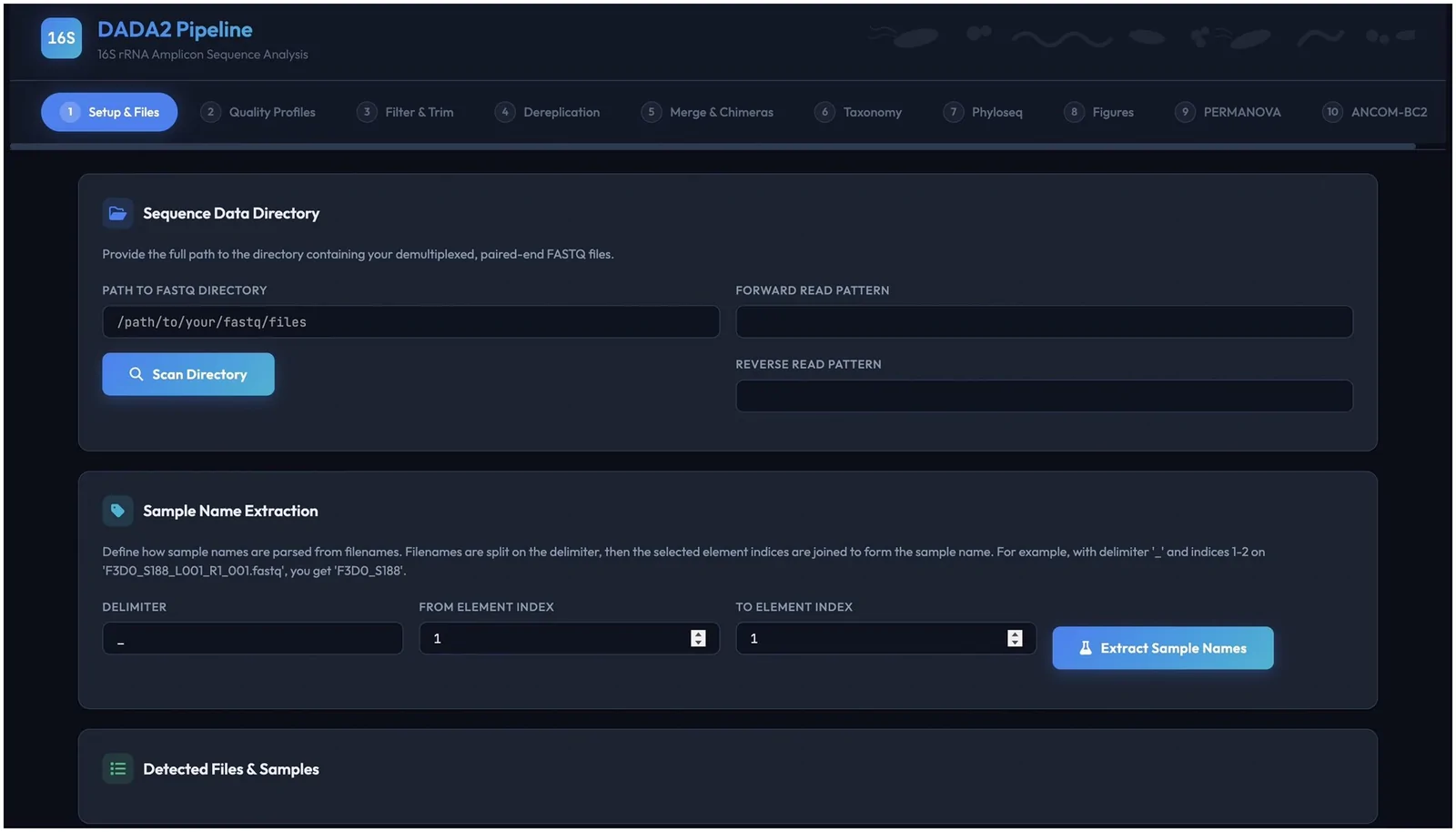
Screenshot of the setup and file detection step of RAPID, showing auto-detected FASTQ file patterns, configurable sample name extraction with live preview, and the scan button.

### 2.3 The ITS application (11 steps)

The ITS application extends the shared framework to an 11-step workflow that addresses the unique requirements of ITS sequencing. The ITS region’s high length variability (200–600 bp) precludes fixed-length truncation and complicates primer removal due to read-through into opposite primers. The workflow comprises: (1) setup and file detection; (2) quality profile visualization; (3) primer removal using cutadapt with automatic N-prefiltering, primer orientation verification, support for multiple primers, and automatic cutadapt detection or installation via pip; (4) quality filtering without truncation but with a configurable minimum length (default 50 bp); (5–6) error learning, dereplication, merging, and chimera removal as in the 16S workflow; (7) taxonomy assignment using UNITE; and (8–11) phyloseq construction, visualization, PERMANOVA, and ANCOM-BC2, identical to the 16S application.

### 2.4 PERMANOVA analysis

Both applications include a dedicated PERMANOVA step using the vegan package (Dixon 2003). Users enter a formula specifying one or more metadata variables (e.g. Treatment + Age), select a distance method (Bray-Curtis, Jaccard, or Euclidean), and configure permutations (default 9999). A live command preview shows the exact R commands. Results include the adonis2 test table, a betadisper homogeneity test with interpretation, and automatic pairwise PERMANOVA with Bonferroni correction when more than two groups are present.

### 2.5 ANCOM-BC2 differential abundance

The final step implements ANCOM-BC2 (Lin and Peddada 2024), which identifies taxa with differential absolute abundances while correcting for sample- and taxon-specific biases. The application uses the raw (untransformed) phyloseq object, as ANCOM-BC2 handles its own bias correction. Users specify fixed and optional random effects formulas, a grouping variable, taxonomic level (default Genus), *P*-value adjustment method, prevalence filter, and library size cutoff. A collapsible advanced settings panel exposes all ANCOM-BC2 parameters including pseudo-count sensitivity analysis, structural zero detection, regularization, iteration and EM controls, mdFDR control, and optional Dunnett’s and trend tests. Results include four downloadable tables: (i) primary analysis showing differentially abundant taxa for each covariate; (ii) global test identifying taxa differing across any groups; (iii) pairwise directional test showing taxa differing between each pair of groups with direction of change; and (iv) Dunnett’s type test comparing each treatment group against a single control.

### 2.6 Data transformation and session persistence

Three data transformations are available post-phyloseq construction: rarefaction (using rarefy_even_depth, default to minimum sample depth), relative abundance, and centered log-ratio (CLR) via the microbiome package, with the latter recommended for Aitchison-distance ordinations. All downstream visualizations and PERMANOVA use the transformed data, while ANCOM-BC2 always operates on raw counts. Pipeline state is automatically saved after each step. On startup, RAPID checks for existing session files and offers to restore them with path validation. The complete R workspace and phyloseq object can be exported for downstream custom analyses.

### 2.7 Runtime and memory scaling

RAPID was benchmarked on a 26 sample 16S dataset (1 671 711 total reads; NCBI SRA BioProject PRJNA1499817) on a 2023 Apple MacBook Pro (M3 Max Chip; 128 GB memory) using four cores. Total wall time was approximately 70 minutes, with taxonomy assignment being the most computationally intensive steps (peak memory: 16.62 GB). Runtime scales approximately linearly with sample count when samples are processed independently (pool = FALSE); users working with larger cohorts should expect proportionally longer runtimes.

### 2.8 Comparison with manual DADA2 run

To verify that RAPID produces results identical to a standard command-line DADA2 workflow, both pipelines were run on the same dataset (PRJNA1499817) using identical parameters, including a fixed random seed (100) applied immediately before taxonomic assignment in each pipeline. Read counts at every processing step—quality filtering, forward and reverse denoising, paired-end merging, and chimera removal—were identical between RAPID and the manual pipeline across all 26 samples (Supplementary Tables 1 and 2, available as supplementary data at *Bioinformatics* online). Both approaches yielded 12 900 ASVs, and taxonomy was assigned identically by both pipelines to 6102 ASVs at the genus level and 1150 at the species level. Bray-Curtis based ordinations (PCoA and NMDS) produced visually indistinguishable sample clustering between the two approaches (Fig. S10, available as supplementary data at *Bioinformatics* online). These results confirm that RAPID reproduces standard DADA2 output exactly when the random seed is controlled, while eliminating the need for command-line interaction.

## 3 Conclusion and outlook

RAPID provides complete, code-free graphical interfaces for both 16S rRNA and ITS amplicon sequence analysis, bridging the gap between the powerful DADA2/phyloseq ecosystem and researchers without programming expertise. Key architectural contributions include asynchronous processing that maintains interface responsiveness during long computations, automatic session persistence for multi-day analyses, guided step-by-step workflows that reduce parameter misconfiguration, and integration of statistical testing within the same interface. All parameters remain fully configurable, ensuring experienced users retain control while novice users benefit from sensible defaults.

Future development directions include arbuscular mycorrhizal fungi analysis, agentic transformation of the apps, phylogenetic tree construction for UniFrac distance calculations, batch processing, additional differential abundance methods, network analysis, and a unified launcher for selecting between 16S and ITS workflows.

## Supplementary Material

btag688_Supplementary_Data

## Data Availability

Raw 16S rRNA amplicon sequences are available in the NCBI Sequence Read Archive under BioProject accession PRJNA1499817. RAPID is freely available at https://github.com/beantkapoor786/RAPID and archived at 10.5281/zenodo.21628564. Both applications can be installed locally on any system with R (≥4.0) and run as local web applications.
