# Supplementary material for: RAPID: an interactive R/Shiny platform for end-to-end 16S rRNA and ITS amplicon sequence analysis using DADA2
Source: Bioinformatics. 2026 Sep 15;42(9):btag688. doi: 10.1093/bioinformatics/btag688 (PMC13619002; doi:10.1093/bioinformatics/btag688)
Supplement: Supplementary file 1 [file btag688_supplementary_data.docx]

**Supplementary Table 1.** Read counts in RAPID pipeline at every processing step (quality filtering, forward and reverse denoising, paired-end merging, and chimera removal) for 26 sample 16S rhizosphere dataset.

| Sample ID | Input reads | Filtered  reads | Denoised forward reads | Denoised reverse reads | Merged reads | Chimeric reads removed |
| --- | --- | --- | --- | --- | --- | --- |
| 1-827-1-1-1-Rhizo-16s | 55,943 | 50,899 | 47,486 | 47,487 | 30,865 | 25,697 |
| 10-405-1-5-1-Rhizo-16s | 66,196 | 58,399 | 54,383 | 54,615 | 34,925 | 29,976 |
| 11-419-1-6-5-Rhizo-16s | 69,810 | 61,947 | 57,783 | 58,185 | 37,926 | 31,384 |
| 12-545-1-7-5-Rhizo-16s | 62,875 | 56,145 | 52,560 | 52,710 | 33,872 | 27,233 |
| 13a-533-1-8-4-Rhizo-16s | 64,436 | 57,883 | 54,483 | 54,329 | 37,500 | 30,639 |
| 14-416-1-8-2-Rhizo-16s | 62,898 | 56,021 | 52,225 | 52,412 | 35,510 | 28,837 |
| 15-437-1-9-2-Rhizo-16s | 71,628 | 65,074 | 61,344 | 61,388 | 43,315 | 33,349 |
| 16-233-2-1-2-Rhizo-16s | 60,172 | 54,446 | 50,435 | 50,975 | 33,369 | 27,027 |
| 17-972-2-1-4-Rhizo-16s | 65,674 | 59,414 | 55,486 | 55,918 | 38,477 | 29,544 |
| 18-567-2-2-2-Rhizo-16s | 59,041 | 52,321 | 49,214 | 48,953 | 34,274 | 27,005 |
| 19-258-2-2-4-Rhizo-16s | 57,934 | 52,512 | 49,027 | 49,066 | 34,538 | 27,321 |
| 20a-543-2-3-5-Rhizo-16s | 52,349 | 46,398 | 43,110 | 43,404 | 28,055 | 21,880 |
| 20b-543-1-4-1-Rhizo-16s | 80,514 | 72,008 | 67,376 | 67,124 | 46,488 | 37,394 |
| 21-206-2-5-1-Rhizo-16s | 73,139 | 65,498 | 61,742 | 61,819 | 43,530 | 32,547 |
| 22-218-2-5-3-Rhizo-16s | 77,008 | 69,381 | 65,556 | 65,104 | 46,107 | 35,728 |
| 23a-601-2-5-4-Rhizo-16s | 60,778 | 54,740 | 51,217 | 51,473 | 34,947 | 28,523 |
| 24a-411-2-6-5-Rhizo-16s | 74,431 | 67,582 | 63,424 | 63,442 | 43,042 | 33,685 |
| 2a-432-1-1-5-Rhizo-16s | 54,359 | 47,906 | 44,716 | 45,016 | 29,912 | 25,916 |
| 3-427-1-2-1-Rhizo-16s | 39,827 | 33,395 | 30,725 | 31,413 | 17,787 | 15,905 |
| 4-342-1-2-2-Rhizo-16s | 50,106 | 44,016 | 40,406 | 41,099 | 25,507 | 21,340 |
| 5-973-1-3-1-Rhizo-16s | 74,842 | 67,352 | 63,145 | 63,138 | 42,342 | 33,064 |
| 6-564-1-3-2-Rhizo-16s | 67,750 | 59,646 | 55,774 | 56,015 | 37,606 | 30,639 |
| 7a-522-1-3-3-Rhizo-16s | 79,055 | 71,636 | 67,543 | 67,816 | 47,775 | 36,129 |
| 8-588-1-3-4-Rhizo-16s | 60,708 | 54,161 | 49,781 | 50,428 | 31,240 | 25,930 |
| 808-2-7-4-Rhizo-16s | 61,756 | 54,730 | 51,007 | 51,312 | 34,323 | 27,638 |
| 9-572-1-4-3-Rhizo-16s | 68,482 | 62,341 | 57,840 | 58,127 | 37,095 | 28,328 |

**Supplementary Table 2.** Read counts in manual DADA2 run at every processing step (quality filtering, forward and reverse denoising, paired-end merging, and chimera removal) for 26 sample 16S rhizosphere dataset.

| Sample | Input reads | Filtered  reads | Denoised forward reads | Denoised reverse reads | Merged reads | Chimeric reads removed |
| --- | --- | --- | --- | --- | --- | --- |
| 1-827-1-1-1-Rhizo-16s | 55,943 | 50,899 | 47,486 | 47,487 | 30,865 | 25,697 |
| 10-405-1-5-1-Rhizo-16s | 66,196 | 58,399 | 54,383 | 54,615 | 34,925 | 29,976 |
| 11-419-1-6-5-Rhizo-16s | 69,810 | 61,947 | 57,783 | 58,185 | 37,926 | 31,384 |
| 12-545-1-7-5-Rhizo-16s | 62,875 | 56,145 | 52,560 | 52,710 | 33,872 | 27,233 |
| 13a-533-1-8-4-Rhizo-16s | 64,436 | 57,883 | 54,483 | 54,329 | 37,500 | 30,639 |
| 14-416-1-8-2-Rhizo-16s | 62,898 | 56,021 | 52,225 | 52,412 | 35,510 | 28,837 |
| 15-437-1-9-2-Rhizo-16s | 71,628 | 65,074 | 61,344 | 61,388 | 43,315 | 33,349 |
| 16-233-2-1-2-Rhizo-16s | 60,172 | 54,446 | 50,435 | 50,975 | 33,369 | 27,027 |
| 17-972-2-1-4-Rhizo-16s | 65,674 | 59,414 | 55,486 | 55,918 | 38,477 | 29,544 |
| 18-567-2-2-2-Rhizo-16s | 59,041 | 52,321 | 49,214 | 48,953 | 34,274 | 27,005 |
| 19-258-2-2-4-Rhizo-16s | 57,934 | 52,512 | 49,027 | 49,066 | 34,538 | 27,321 |
| 20a-543-2-3-5-Rhizo-16s | 52,349 | 46,398 | 43,110 | 43,404 | 28,055 | 21,880 |
| 20b-543-1-4-1-Rhizo-16s | 80,514 | 72,008 | 67,376 | 67,124 | 46,488 | 37,394 |
| 21-206-2-5-1-Rhizo-16s | 73,139 | 65,498 | 61,742 | 61,819 | 43,530 | 32,547 |
| 22-218-2-5-3-Rhizo-16s | 77,008 | 69,381 | 65,556 | 65,104 | 46,107 | 35,728 |
| 23a-601-2-5-4-Rhizo-16s | 60,778 | 54,740 | 51,217 | 51,473 | 34,947 | 28,523 |
| 24a-411-2-6-5-Rhizo-16s | 74,431 | 67,582 | 63,424 | 63,442 | 43,042 | 33,685 |
| 2a-432-1-1-5-Rhizo-16s | 54,359 | 47,906 | 44,716 | 45,016 | 29,912 | 25,916 |
| 3-427-1-2-1-Rhizo-16s | 39,827 | 33,395 | 30,725 | 31,413 | 17,787 | 15,905 |
| 4-342-1-2-2-Rhizo-16s | 50,106 | 44,016 | 40,406 | 41,099 | 25,507 | 21,340 |
| 5-973-1-3-1-Rhizo-16s | 74,842 | 67,352 | 63,145 | 63,138 | 42,342 | 33,064 |
| 6-564-1-3-2-Rhizo-16s | 67,750 | 59,646 | 55,774 | 56,015 | 37,606 | 30,639 |
| 7a-522-1-3-3-Rhizo-16s | 79,055 | 71,636 | 67,543 | 67,816 | 47,775 | 36,129 |
| 8-588-1-3-4-Rhizo-16s | 60,708 | 54,161 | 49,781 | 50,428 | 31,240 | 25,930 |
| 808-2-7-4-Rhizo-16s | 61,756 | 54,730 | 51,007 | 51,312 | 34,323 | 27,638 |
| 9-572-1-4-3-Rhizo-16s | 68,482 | 62,341 | 57,840 | 58,127 | 37,095 | 28,328 |

**Supplementary Figure S1.** Screenshot of the filter and trim step with configurable parameters and sensible defaults based on standard DADA2 16S workflow.

**Supplementary Figure S2.** Screenshot of the DADA2 error learning algorithm and dereplication step.

**Supplementary Figure S3.** Screenshot of the merged read pairs and remove chimera step.

**Supplementary Figure S4.** Screenshot of the read tracking table and line graph for each sample.

**Supplementary Figure S5.** Screenshot of the assignTaxonomy step using Bayesian or IDTAXA classifier algorithm.

**Supplementary Figure S6.** Screenshot of the Phyloseq step where users upload metadata file for phyloseq object construction.

**Supplementary Figure S7.** Downloaded plots of (A) Rarefaction (B) box plot of alpha diversity indices (Observed, Shannon, and Simpson), (C) NMDS ordination plot, and (D) PCoA ordination plot.

**Supplementary Figure S8.** Screenshot of the PERMANOVA step implemented using the vegan package.

**Supplementary Figure S9.** Screenshots of the ANCOM-BC2 (A) command set-up and (B) primary analysis result for the differential abundance analysis at every taxa.

**Supplementary Figure S10.** PCoA ordinations generated by (A) RAPID and (B) manual DADA2 run. NMDS ordinations generated by (C) RAPID and (D) manual DADA2 run on 26 sample 16S rhizosphere dataset.
